# Advances in Confocal Microscopy of the Eye

**DOI:** 10.1155/2016/1794240

**Published:** 2016-05-26

**Authors:** Paolo Fogagnolo, Michele Iester, Hong Liang, Dipika V. Patel

**Affiliations:** ^1^Eye Clinic, Ospedale San Paolo, Dipartimento Testa-Collo, Università degli Studi, Milan, Italy; ^2^Anatomical-Clinical Laboratory for Functional Diagnosis and Treatment of Glaucoma and Neuroophthalmology, Eye Clinic, DiNOGMI, University of Genoa, Genoa, Italy; ^3^Department of Ophthalmology III, Quinze-Vingts National Ophthalmology Hospital, Paris, France; ^4^Department of Ophthalmology, New Zealand National Eye Centre, Faculty of Medical and Health Sciences, University of Auckland, Auckland, New Zealand

This special issue focuses on the recent advances on* in vivo *confocal microscopy (IVCM), a technique used to investigate eye structures at the cellular level without tissue damage.

In 1985 Lemp and coworkers were the first to study* ex vivo* corneas by confocal microscopy and to suggest a possible* in vivo* use [1]. In the early 1990s, the groups directed by Cavanagh and Kaufman published the first IVCM in humans; thereafter the scientific interest in ocular IVCM rapidly increased. In the last decade, IVCM progressively gained a relevant role in the clinical setting, being of help in the diagnosis and management of a number of conditions such as toxicity induced by preservatives [2, 3] and different eye treatments [4, 5], iatrogenic damage [6], infections [7], and dystrophies [8], pathology of the conjunctiva [9–11] and limbus [12], ocular surface tumors [13], and corneal deposits [14, 15].

IVCM is a valuable tool for enhancing our understanding of anterior segment physiology and pathology, as pointed out by Dr. V. Fasanella et al. in their paper investigating meibomian gland changes occurring with aging and a number of ocular surface diseases.

Examples of the usefulness of IVCM in detecting early corneal changes can be found in the paper by Dr. D. Wang et al., who highlighted that a higher-than-normal immune activity may be observed in a subgroup of patients with clear corneal grafts in the absence of any other clinical signs. Dr. P. Song et al. studied the confocal and histopathological features of the stromal scar in keratoconus and suggested that IVCM is capable of detecting subtle keratocyte activation associated with fibrosis.

In recent years, IVCM has become increasingly useful in evaluating corneal innervation (which is of key importance in regulating ocular surface homeostasis) [16] and corneal immune and inflammatory responses [17]. In this special issue, three papers explored the changes occurring in corneal innervation in both local and systemic diseases. The paper by Dr. E. F. Wang et al. reviewed the impact of various systemic diseases on corneal innervation and discussed the potential use of IVCM as a noninvasive marker of peripheral neuropathy. The reports by Dr. R. Shetty et al. and Dr. N. K. Pahuja et al. investigated nerve changes occurring, respectively, in dry eye disease and keratoconus.

Finally, IVCM plays a role in studying the therapeutic effects of topical eye treatments, as shown in this issue by the paper of Dr. A. M. Fea et al.

This special issue provides a useful update on the advances in the rapidly evolving field of IVCM and highlights the contribution of this technology to our understanding of the anterior segment in health and disease.

*Paolo Fogagnolo* *Michele Iester* *Hong Liang* *Dipika V. Patel*
